# Supplementary material for: Context-aware single-cell multiome approach identified cell-type specific lung cancer susceptibility genes
Source: bioRxiv. 2023 Sep 26:2023.09.25.559336. Preprint. [Version 1] doi: 10.1101/2023.09.25.559336 (PMC10557605; doi:10.1101/2023.09.25.559336)
Supplement: 1 [file NIHPP2023.09.25.559336V1-supplement-1.pdf]

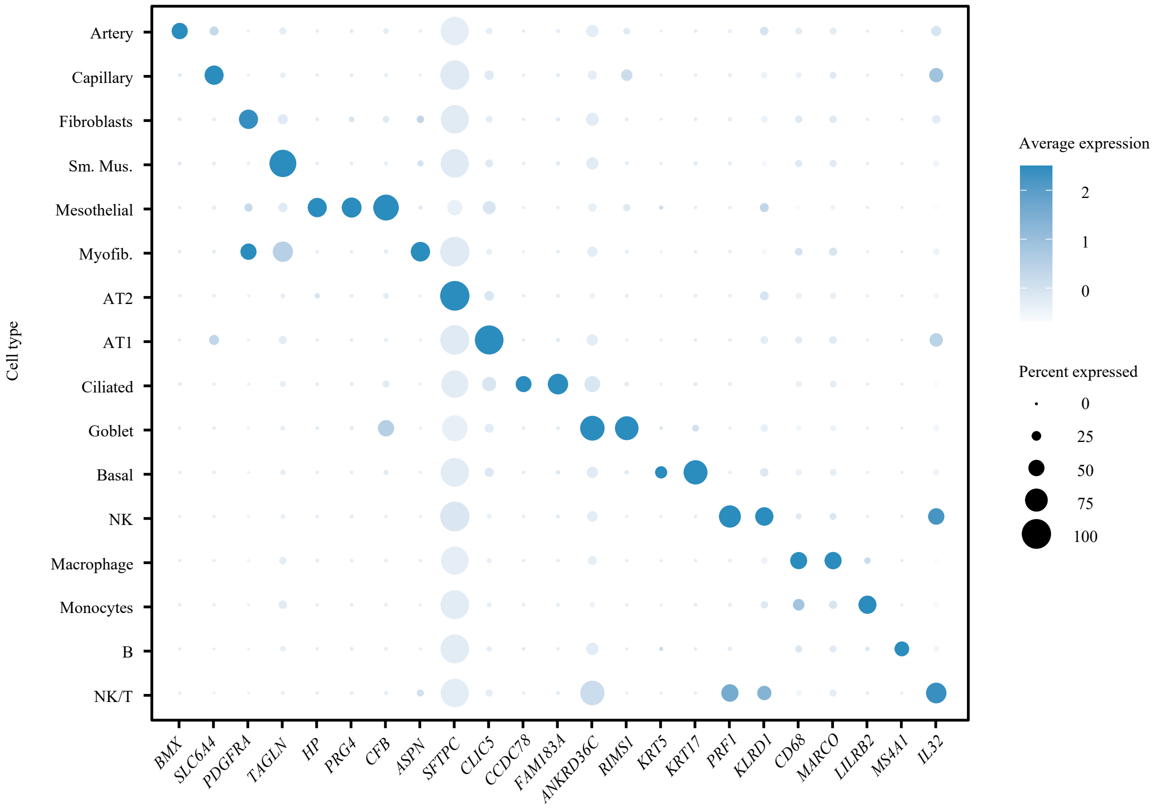

Figure S1. Additional canonical markers for cell-type annotation. Dot plot visualizing the normalized RNA expression of selected marker genes by cell type. The color and size of each dot correspond to the scaled average expression level and fraction of expressing cells, respectively.

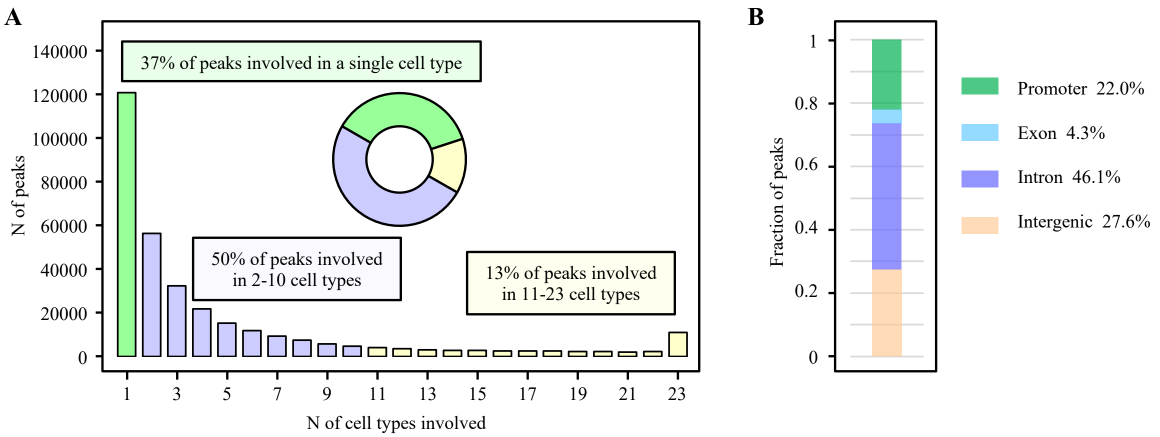

Figure S2. Accessible chromatin peaks identified from snATAC-seq data across different cell types. (A) Y-axis refers to number of peaks and X-axis refers to number of cell types involved. The piechart represents the proportion of peaks in single, 2-10, or 11-23 cell types. **(B)** Fraction of peaks were annotated into different categories (promoter, exon, intron, or intergenic).

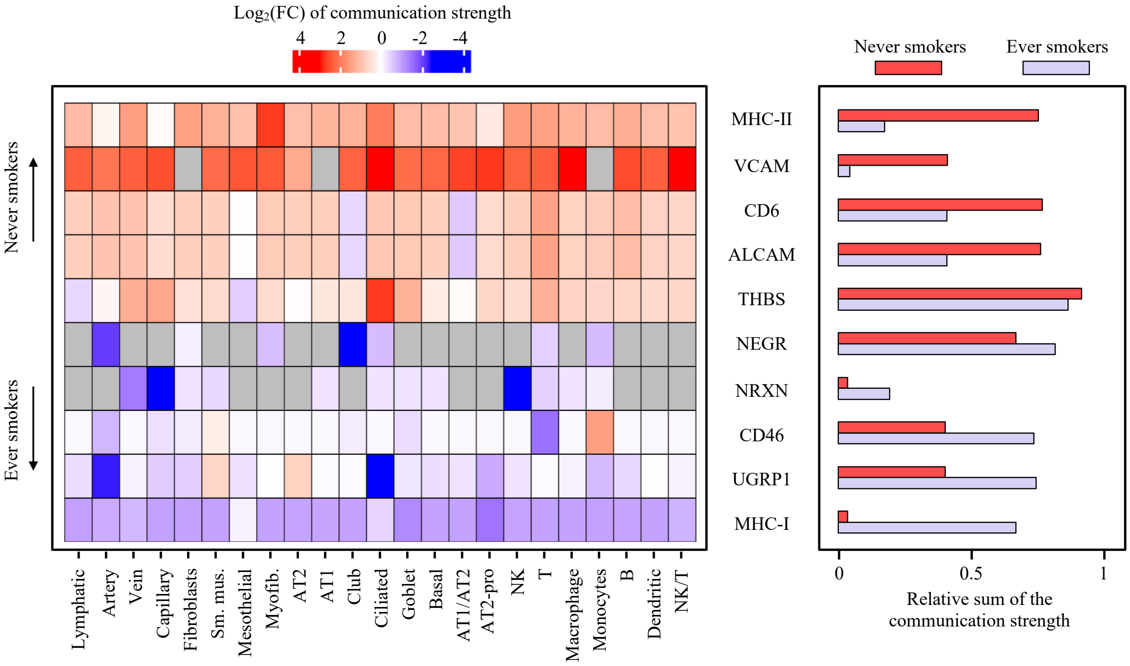

Figure S3. Differential intercellular communication based on smoking status. Heatmap of the top 5 elevated pathways in ever- (blue) and never-smokers (red) across cell types are shown on the left. Color indicates the log-transformed ratio of pathway-level communication strength between ever- and never-smokers (relative to never-smokers). Gray indicates that the pathway strength is 0 in the cell types of smokers and never smokers. The right part presents the summarized communication strength of each pathway for ever- (blue) and never-smokers (red).

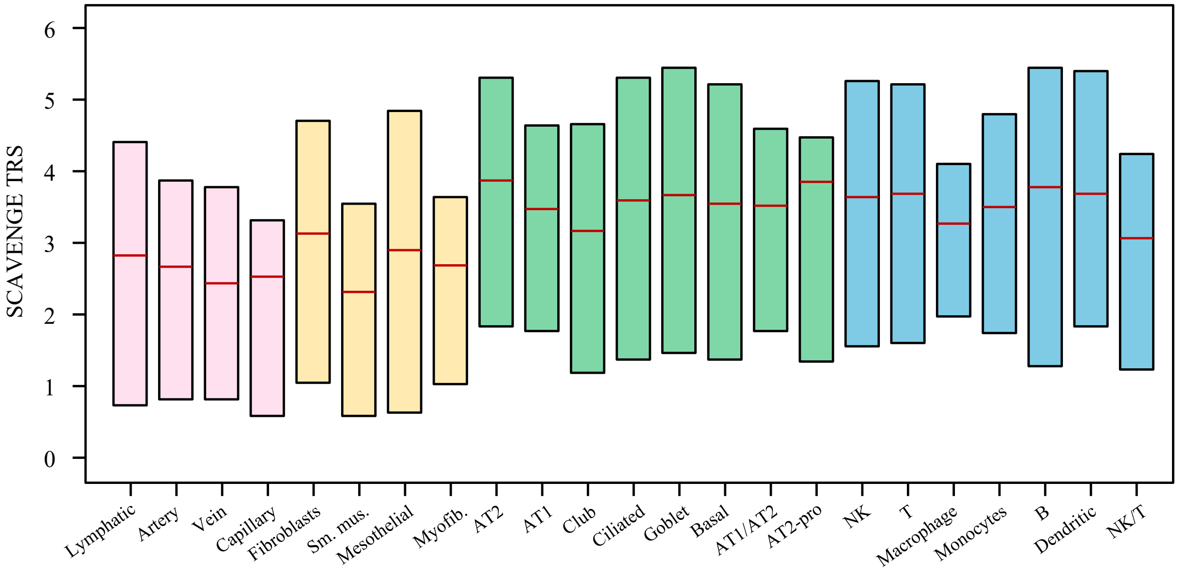

Figure S4. Trait relevance scores across cell types. The distribution of trait relevance scores (TRS) of lung cancer risk across cell types (endothelial in red, stromal in yellow, epithelial in green, and immune in blue) were presented as a boxplot. The red horizonal band shows the mean score, and the box indicates the middle 50% of cells.

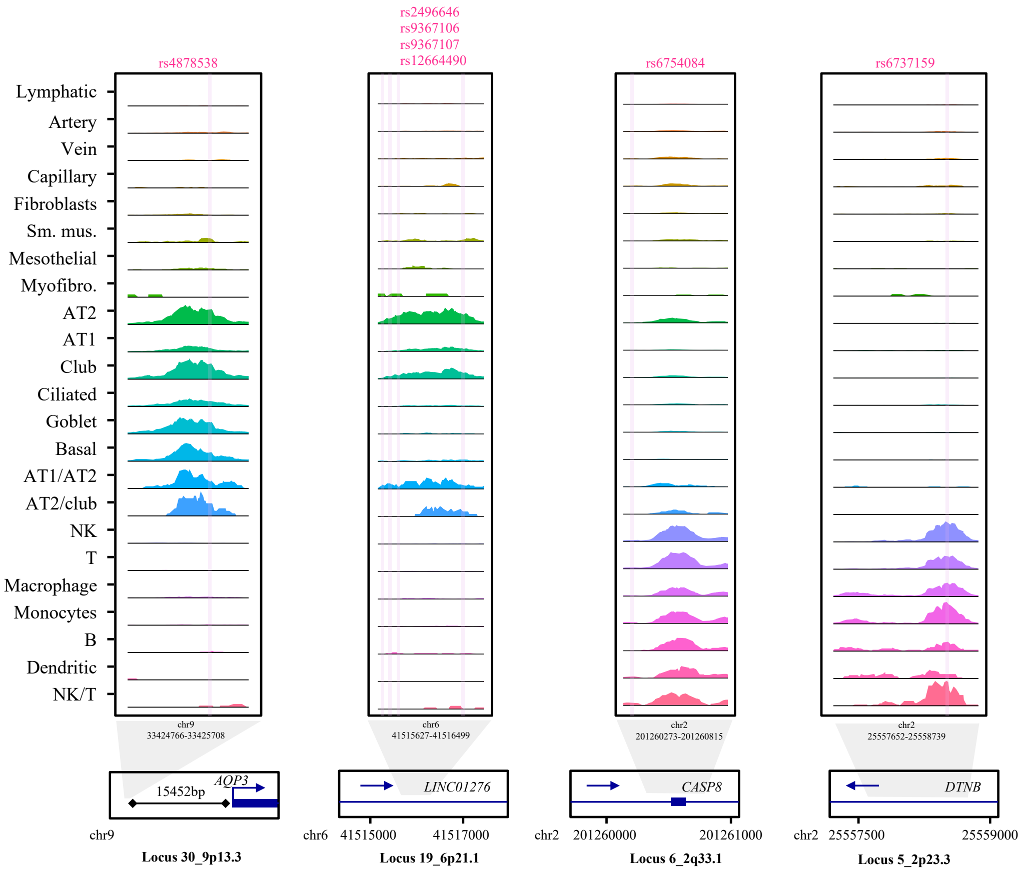

Figure S5. Epithelial or immune cell specific CCV-overlapping cCREs. The sequencing tracks representing chromatin accessibility of four different loci are displayed (locus IDs at the bottom). The rsIDs of CCVs are shown above the tracks and marked with vertical pink lines to indicate their genomic positions. Each track represents the aggregated snATAC signal of all cell types, normalized by the total number of reads in the regions (normalized values from left to right: 0-180, 0-180, 0-500, 0-240). Arrows depict the transcriptional directions of each gene.

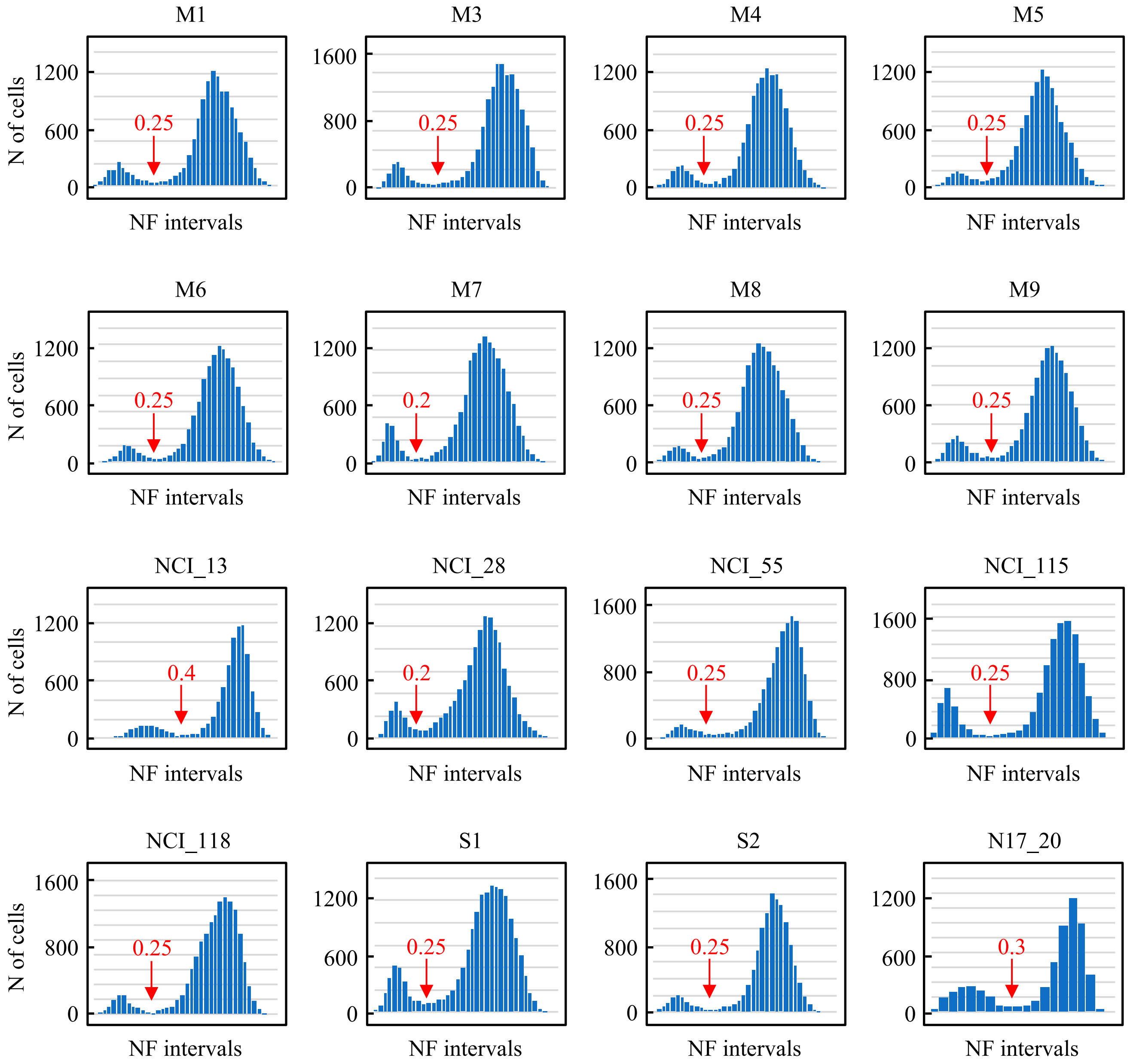

Figure S6. Determination of the cutoff in defining the empty droplet using DropletQC. The nuclear fraction (NF) of each cell is calculated by DropletQC. Empty droplets were identified by visualizing the density of nuclear fraction and setting the cutoff according to the “peak” in low-nuclear-fraction droplets.
